# Lack of Cannabinoid Receptor Type-1 Leads to Enhanced Age-Related Neuronal Loss in the Locus Coeruleus

**DOI:** 10.3390/ijms22010005

**Published:** 2020-12-22

**Authors:** Alessandra Gargano, Eva Beins, Andreas Zimmer, Andras Bilkei-Gorzo

**Affiliations:** 1 Institute of Molecular Psychiatry, Medical Faculty, University of Bonn, Venusberg-Campus 1, 53127 Bonn, Germany; gargano@uni-bonn.de (A.G.); e.beins@uni-bonn.de (E.B.); a.zimmer@uni-bonn.de (A.Z.); 2 Institute of Human Genetics, Medical Faculty, University of Bonn, Venusberg-Campus 1, 53127 Bonn, Germany

**Keywords:** endocannabinoid system, stereological counting, noradrenergic neurons, ageing

## Abstract

Our laboratory and others have previously shown that cannabinoid receptor type-1 (CB1r) activity is neuroprotective and a modulator of brain ageing; a genetic disruption of CB1r signaling accelerates brain ageing, whereas the pharmacological stimulation of CB1r activity had the opposite effect. In this study, we have investigated if the lack of CB1r affects noradrenergic neurons in the locus coeruleus (LC), which are vulnerable to age-related changes; their numbers are reduced in patients with neurodegenerative diseases and probably also in healthy aged individuals. Thus, we compared LC neuronal numbers between cannabinoid 1 receptor knockout (*Cnr1*^−/−^) mice and their wild-type littermates. Our results reveal that old *Cnr1*^−/−^ mice have less noradrenergic neurons compared to their age-matched wild-type controls. This result was also confirmed by the analysis of the density of noradrenergic terminals which proved that *Cnr1*^−/−^ mice had less compared to the wild-type controls. Additionally, we assessed pro-inflammatory glial activity in the LC. Although the density of microglia in *Cnr1*^−/−^ mice was enhanced, they did not show enhanced inflammatory profile. We hypothesize that CB1r activity is necessary for the protection of noradrenergic neurons, but its anti-inflammatory effect probably only plays a minor role in it.

## 1. Introduction

The locus coeruleus (LC), or “blue spot”, is a small nucleus located in the pons of the brainstem, lateral to the IVth ventricle. It projects throughout the neuroaxis and represents the major source of noradrenaline (NE) in the central nervous system. Although the LC consists of a relatively low number of noradrenergic neurons (45–50 thousand in a normal healthy young adult human [1] and approximately 1500 in adult mice [2]), its projecting area is extremely wide; noradrenergic terminals are abundantly present in the cortex, mainly in the somatosensory and motor cortex [3], hippocampus and amygdala [3,4,5], hypothalamus [6] and in the brainstem itself [7]. The LC also receives a large number of afferents, mainly from the cortex, amygdala [8,9] and from the spinal cord [10].

Electrophysiological studies revealed that LC noradrenergic neurons have a peculiar double firing mode; they are able to switch between tonic or phasic mode, regulating different behavioral states. A tonic firing is related to a “low attention” mode, while the switch to the phasic firing happens in response to a relevant “focus-demanding” stimuli [11]. These studies suggested that LC may play a role in modulating numerous cognitive functions, including attention, memory as well as in the regulation of sleep–wake states and stress response [12]. Under physiological conditions, LC neurons respond to external and internal sensory stimuli and this response influences learning and memory processes [13]. Electrophysiological recording studies showed that LC activation induced hippocampal long-term depression, which was dependent on adrenergic receptor activation [14,15]. Moreover, a depletion of LC neurons impairs working memory and hippocampal neurogenesis, also suggesting a direct link between LC functionality and hippocampal-related control of cognition [16]. Besides lesion, pharmacological manipulation of LC can also significantly affect cognitive processes [17,18].

Interest related to the LC-noradrenaline system emerged particularly in aging research. Studies revealed that the noradrenergic system can influence the process of brain ageing and that it also changes during ageing. It was reported that NE has a neuroprotective effect against inflammation and excitotoxicity. Increasing NE levels in vivo improved cognition while in vitro NE administration protected neurons against β-amyloid toxicity. On the other side, the LC is vulnerable to age-related changes and particularly impacted in the most common neurodegenerative diseases, Alzheimer (AD) and Parkinson (PD) diseases [19].

Most strikingly, in AD patients, the neuronal loss in the LC is higher than the loss of cholinergic neurons in the nucleus basalis (67.9% vs. 41.1%) [20] and also in PD the neuronal loss is more intensive in the LC (83.2%) than in the substantia nigra (77.8%) [21].

While it seems well established that dysfunctions of the noradrenergic system are correlated with neurodegenerative diseases, the question whether or not LC neurons are also lost during normal physiological ageing has not been answered conclusively [22]. Some postmortem studies have reported an age-related neurodegeneration in LC neuron number of about 20–40% [23], whereas other postmortem studies using unbiased stereological counting and strictly excluding samples with pathological changes (like neurofibrillary tangles) did not find a reduction of LC cell number in healthy adults [24,25]. Nevertheless, a recent in vivo study based on a sophisticated magnetization transfer weighted imaging technique showed a relationship between LC signal intensity values and age, revealing an age-related decline in LC signal intensity values from the age of 60 confined to the rostral portion of the LC. This finding thus supports an age-related shrinkage or loss of neuromelanin containing noradrenergic neurons in the LC also in healthy subjects [26]. Whether an age-related decline in the number of LC neurons is present in mice is still controversial, ranging from a decline [27] to no change or even an increase in neuronal numbers [28,29]. These discrepancies may be attributed in part to the fact that different mouse strains have been used.

There is a large body of evidence demonstrating that cannabinoid receptor type-1 (CB1r) signaling modulates the activity of the LC. First of all, the immunoreactivity for CB1r within the LC was localized in somatodendritic structures, axon terminals, and also on some glial processes [30]. In the frontal cortex, one of the main projection areas of the LC, CB1 receptors were also identified on noradrenergic axon terminals. CB1r activity can influence both inhibitory and excitatory signaling, although most of the axonal CB1 receptors in the LC are on inhibitory and only a minority on excitatory synapses [30]. Therefore, it is not surprising that systemic administration of the CB1r agonist WIN55,212-2 has been shown to increase the firing frequency of noradrenergic neurons and thus to enhance forebrain NE release [31,32]. In further support, CB1r deletion caused significant alterations of the electrophysiological properties of noradrenergic neurons such as an increase in LC-NE excitability and input resistance. Moreover, the increase in LC-NE excitability observed in wild-type mice following CRF application was not observed in CB1r knockout (*Cnr*^−/−^) mice. These results indicate that CB1r deletion causes a disruption in LC-NE signaling, proving a basal endocannabinoid regulation of LC-NE activity [33].

Cannabinoid system activity not only regulates neuronal activity but may also influence their survival. *Cnr1*^−/−^ mice show a loss of principal neurons in the hippocampus [34] accompanied by histological signs of brain ageing such as reduced neurogenesis and neuroinflammation [35] as well as an enhanced accumulation of the ageing pigment lipofuscin [36].

In the present study, we asked whether CB1r activity, similar to that in hippocampus, influences neuronal survival and neuroinflammation in the LC during ageing.

## 2. Results

### 2.1. Enhanced Age-Related Neuronal Loss in the Locus Coeruleus in Cnr1^−/−^ Mice

**Figure 1:**
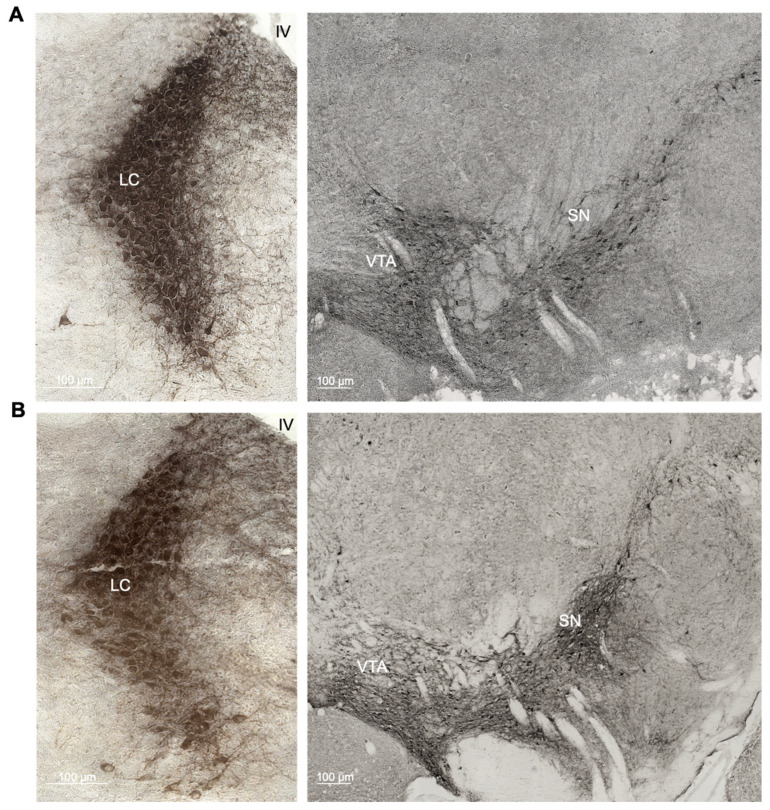

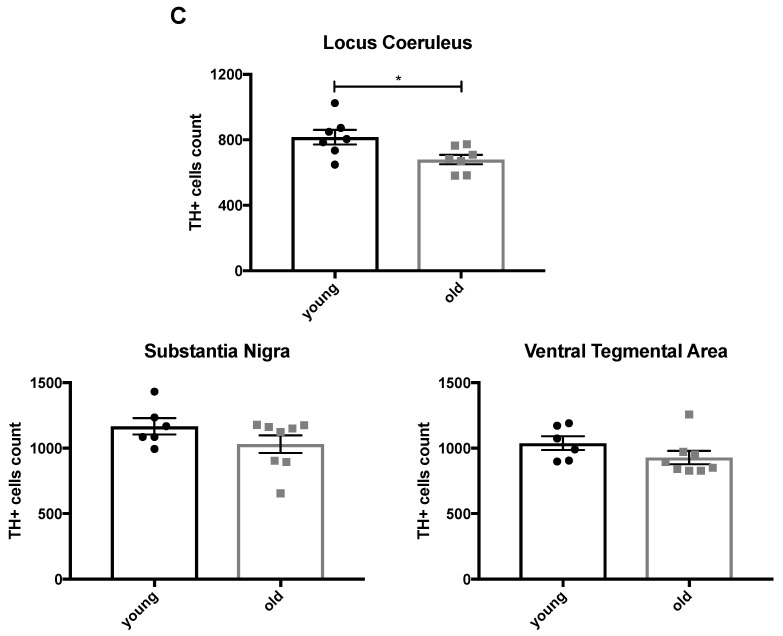
(**A**,**B**) Representative photomicrographs of the tyrosine hydroxylase (TH)-positive regions locus coeruleus (LC), substantia nigra (SN) and ventral tegmental area (VTA) in young and old animals. (**C**) Quantitative stereological analysis of the total number of TH-positive cells of young (3-month-old) and old (22-month-old) C57BL/6J wild-type mice.* *p* < 0.05 according to Student’s *t*-test (*n* = 6–8 per age group). Dots represent single animals, columns represent mean values, and error bars represent standard error of means (SEM).

To determine if there is an age-related neuronal loss in the catecholaminergic nuclei in C57BL/6J mice, we compared the number of tyrosine hydroxylase (TH)-positive cells in the locus coeruleus (LC), substantia nigra (SN) and ventral tegmental area (VTA) between 3- (Figure 1A) and 22-month-old wild-type mice by stereological counting (Figure 1B). The number of TH-positive cells in the LC was significantly reduced in old compared to young C57BL/6J mice (t_10_ = 2.663; *p* = 0.0238), whereas neither the SN (t_12_ = 1.425; *p* = 0.1798) nor the VTA (t_12_ = 1.483; *p* = 0.1639) showed significant differences between the age groups (Figure 1C).

**Figure 2:**
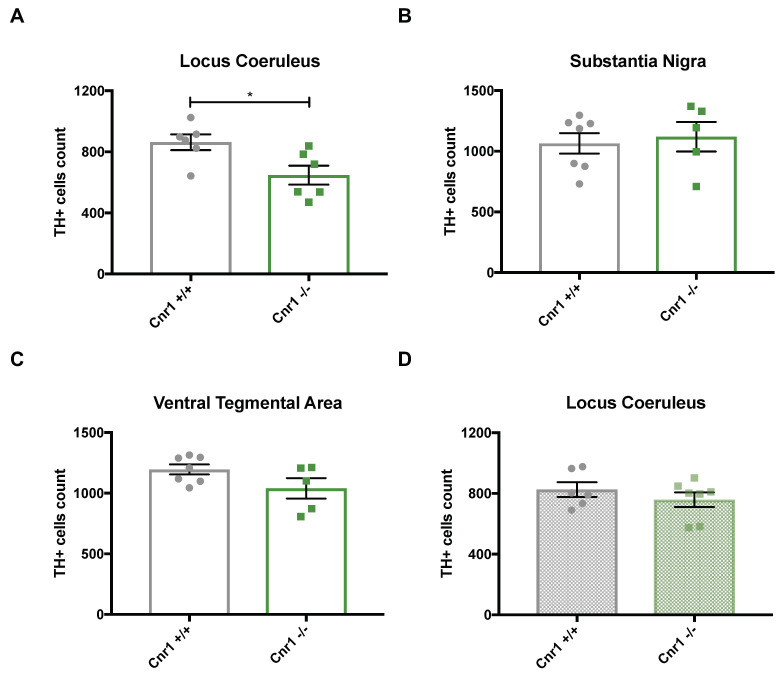
Quantitative stereological analysis of the total number of tyrosine hydroxylase (TH)-positive cells in 18-month-old *Cnr1*^+/+^ wild-type and *Cnr1*^−/−^ animals within the (**A**) locus coeruleus, (**B**) substantia nigra, (**C**) ventral tegmental area and (**D**) in 3-month-old *Cnr1*^+/+^ wild-type and *Cnr1*^−/−^ animals within the locus coeruleus.* *p* < 0.05 according to Student´s *t*-test (*n* = 5–7 per genotype). Dots represent single animals, columns represent mean values, error bars represent standard error of means (SEM).

Subsequently, we compared the number of TH-positive cells in the LC, SN and VTA between 18-month-old wild-type and *Cnr1*^−/−^ mice. In the LC, the number of TH-positive cells was significantly lower in *Cnr1*^−/−^ mice than in age-matched wild-type littermates (t_10_ = 2.663; *p* = 0.0238) (Figure 2A). In contrast, we found no genotype effects for the VTA (t_10_ = 1.806; *p* = 0.1010) or the SN (t_10_ = 0.385; *p* = 0.7084) (Figure 2B,C). To test whether *Cnr1*^−/−^ mice generally have a reduced number of LC neurons independently from their age, we also analyzed the number of TH-positive cells in the LC in 3-month old wild-type and *Cnr1*^−/−^ mice (Figure 2D). We found no difference between the genotypes (t_11_ = 0.971; *p* = 0.352), thus strongly indicating that the difference observed in old mice is due to an exacerbated age-related loss of TH-positive LC neurons in *Cnr1*^−/−^ mice.

Note that only groups represented on the same panels in Figure 1; Figure 2 are comparable, because they were stained together in the same staining series. As staining intensity varies between series, results are not comparable between different figures or panels.

### 2.2. Reduced Density of Noradrenergic Terminals in Aged Cnr1^−/−^ Mice

**Figure 3:**
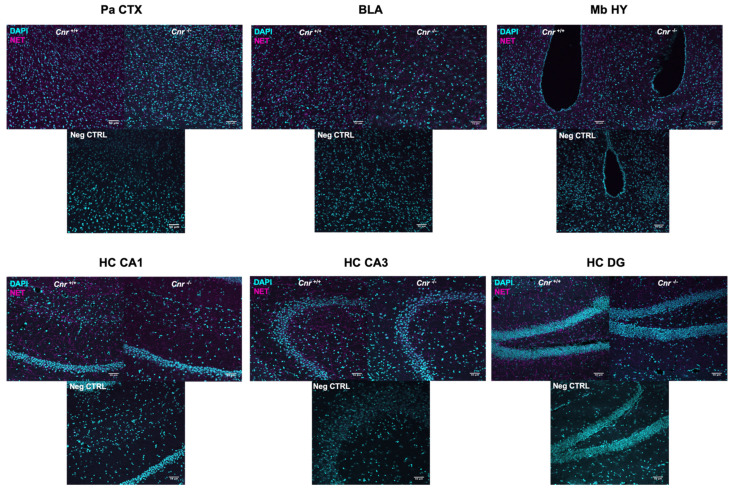
Representative photomicrograph of norepinephrine transporter (NET) staining within the parietal cortex (Pa CTX), basolateral amygdala (BLA), mediobasal hypothalamus (Mb HY) and hippocampal cornu ammonis 1 (CA1), cornu ammonis 3 (CA3), and dentate gyrus (DG) regions of 18-month-old wild-type (left) and *Cnr1*^−/−^ (right) animals. Negative controls (Neg CTRL)were stained only with the secondary antibodies. Scalebar: 50 µM.

We next wished to determine the density of noradrenergic terminals. For this purpose, we analyzed the area covered by the norepinephrine transporter (NET) in wild-type and *Cnr1*^−/−^ mice across the following regions of the main output areas of the LC: parietal cortex (Pa CTX), basolateral amygdala (BLA), mediobasal hypothalamus (Mb HY) and CA1, CA3 and dentate gyrus (DG) regions of the hippocampus (HC) (Figure 3).

**Figure 4:**
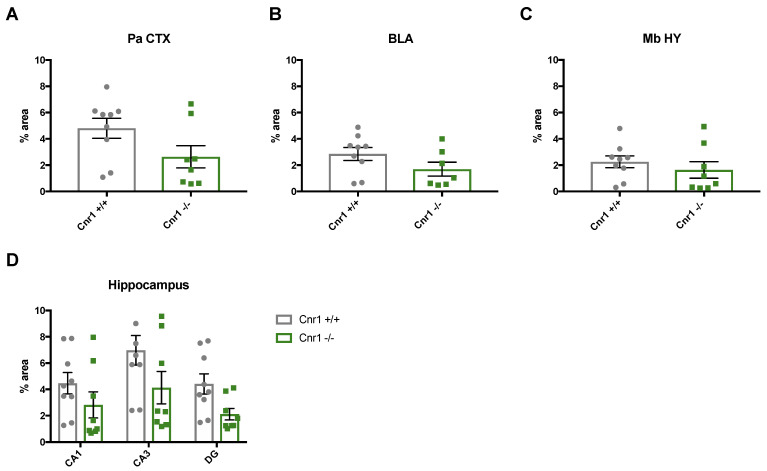
Analysis of the density of adrenergic axon terminals are covered by NET-positive signal intensity in the parietal cortex (Pa CTX), basolateral amygdala (BLA), mediobasal hypothalamus (Mb HY) and hippocampal cornu ammonis 1 (CA1), cornu ammonis 3 (CA3), and dentate gyrus (DG) regions of 18-month old *Cnr1*^+/+^ and *Cnr1*^−/−^ animals. N = 7–9 per genotype; Dots represent single animals, columns represent mean values, error bars represent standard error of means (SEM).

Our data show that the area covered by NET-positive signal was significantly lower in 18-month-old *Cnr1*^−/−^ mice (genotype effect: F_(1,15)_ = 8.104; *p* = 0.0122) in all the target regions (region x genotype interaction F_(5,750)_ = 0.6898; *p* = 0.6327) (Figure 4).

The density of the NET-positive signal differed significantly between the target regions (F_(5,75)_ = 5.964; *p* = 0.0001), being the lowest in the Mb HY and the highest in the CA3 region of the hippocampus. There was no genotype effect in young (3-month-old) mice (F_(1,9)_ = 0.005; *p* = 0.945) and no genotype x region interaction (F_(5,45)_= 0.130; *p* = 0.985).

**Figure 5:**
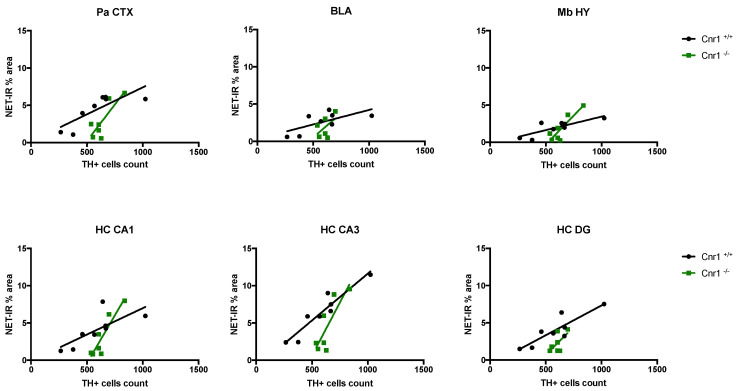
Correlation analysis between the number of tyrosine hydroxylase (TH)-positive cells and the NET signal intensity parietal cortex (Pa CTX), basolateral amygdala (BLA), mediobasal hypothalamus (Mb HY) and hippocampal cornu ammonis 1 (HC CA1), cornu ammonis 3 (HC CA3), and dentate gyrus (HC DG) in wild-type and *Cnr1*^−/−^ mice.

Importantly, we found a positive correlation between the number of TH-positive cells and the NET-positive axon densities in both genotypes in the Pa CTX, Mb HY, CA1 and CA3 regions: lower cell numbers were associated with reduced NET densities (Figure 5 and Table 1). Nevertheless, steady-state noradrenaline levels were not different between the genotypes in any of the brain regions tested: Pa CTX (t_10_ = 0.275; *p* = 0.789), BLA (t_10_ = 0.787; *p* = 0.450), Mb HY (t_9_ = 1.694; *p* = 0.125), HC (t_10_ = 0.776; *p* = 0.4554).

### 2.3. Enhanced Microglia Densities in the LC of Old Cnr1^−/−^ Mice

**Figure 6:**
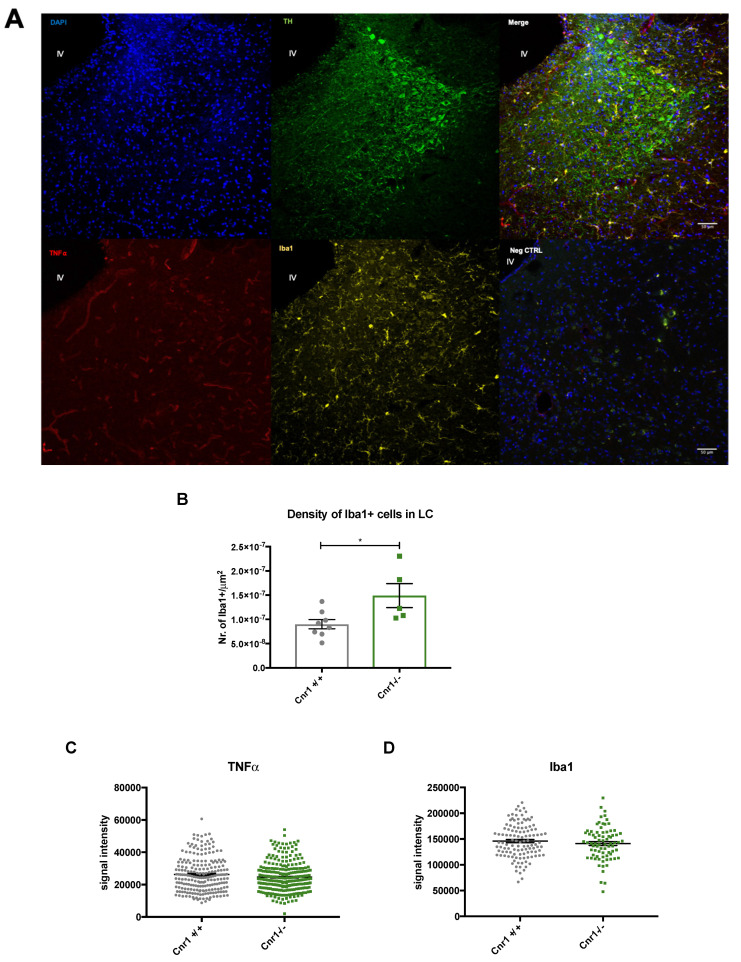

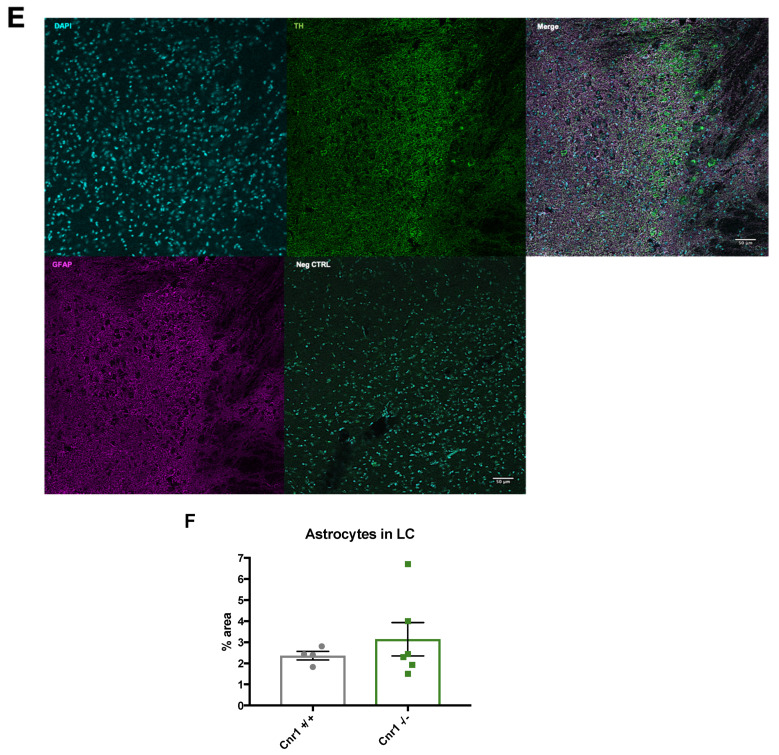
(**A**) Representative photomicrograph of tumor necrosis factor (TNF⍺), tyrosine hydroxylase (TH) and ionized calcium-binding adapter molecule 1 (Iba1) staining within the LC of an 18-month-old wild-type animal. Negative controls (Neg CTRL) were stained only with the secondary antibodies. Scalebar: 50 µM. (**B**) Density of Iba1-positive microglia within the LC. * *p* < 0.05 according to Student´s *t*-test (*n* = 5–8 per genotype). Dots represent single animals, columns represent mean values, error bars represent standard error of means (SEM). (**C**) Analysis of TNF⍺ and (**D**) Iba1 signal intensity in microglia cells within the LC. Dots represent individual values (*n* = 224–291 per genotype). (**E**). Representative photomicrographs of glial fibrillary acidic protein (GFAP) and tyrosine hydroxylase (TH) staining in the LC of an 18-month-old wild-type mouse. Negative controls (Neg CTRL) were stained only with the secondary antibodies. Scalebar: 50 µM. (**F**) Percentage of area covered by GFAP signal within LC (*n* = 4–6 per genotype). Dots represent single animals, columns represent mean values, error bars represent standard error of means (SEM).

To assess inflammatory markers within the LC of 18-month-old wild-type and *Cnr1*^−/−^ mice, we investigated the density of ionized calcium-binding adapter molecule 1 (Iba1)-positive microglia, the level of tumor necrosis factor (TNFα) and the area covered by glial fibrillary acidic protein (GFAP)-positive astrocytes. In *Cnr1*^−/−^ animals, there was a marked increase (+66.2%) in microglia density within the TH-positive area (t_11_ = 2.602; *p* = 0.0246) (Figure 6A,B). However, we did not find any genotype differences in other neuroinflammation markers: Iba1 (t_192_= 0.984; *p* = 0.326), TNFα (U = 28924; *p* = 0.167), GFAP (U = 11; *p* = 0.914) (Figure 6C–F).

## 3. Discussion

The findings from this study strongly indicate that a constitutive genetic disruption of CB1r signaling accelerates the age-related loss of noradrenergic LC neurons in mice of the C57BL/6J genetic background. Thus, using unbiased stereological counting now we found less noradrenergic neurons in the LC of old C57BL/6J mice compared to young ones, whereas the number of dopaminergic neurons in the SN (and also in the VTA) remained unchanged. Even though the results from similar analyses in different mouse strains were contradictory, a reduction in noradrenergic neurons in C57BL/6J mice was also reported in an earlier study. The authors reported an even more dramatic decline in cell numbers [27], which may be related to the fact that they did not use a stereological technique. The age-related loss of LC neurons was more pronounced upon deletion of the CB1 receptor in *Cnr1*^−/−^ mice. In old *Cnr1*^−/−^ mice, the number of LC neurons, but not SN or VTA neurons, was lower than in age-matched wild-type controls. This difference is not due to a developmental effect of the CB1 receptor deletion, because there was no genotype effect in LC neuronal numbers in young animals. Our findings are in line with the previously reported enhanced age-related loss of hippocampal neurons in *Cnr1*^−/−^ mice [34]. It is important to note, however, that *Cnr1*^−/−^ mice do not show a general decline in neuronal numbers in old animals. Rather, they seem to be restricted to the LC and hippocampus, which are brain areas that show neuronal loss during ageing in wild-type animals. Further studies using multiple age groups can answer the question as to whether the reason for the reduced LC neuronal numbers in old *Cnr1*^−/−^ mice is an earlier onset of the neuronal death or the neuronal loss being more intensive.

In general, neuronal numbers are largely preserved during ageing in most of the brain areas, but in several regions, like in the SN [37] or in the LC [22], a significant neuronal loss was detected in the elderly. A significant—28%—loss of SN dopaminergic neurons was detected in mentally healthy older adults [38], whereas, in Parkinson´s disease, the neuronal loss is much more severe (66% compared to age-matched controls) [37]. A significant reduction in LC noradrenergic neurons is present in Alzheimer´s disease from the early phases of the disease onward [39]. It is still not entirely clear if normal, healthy ageing is also associated with a moderate loss of noradrenergic neurons or if symptom-free individuals with a reduced number of LC neurons are in the prodromal, symptom-free phase of the disease. A reduced noradrenergic signaling can contribute not only to the pathogenesis of Alzheimer´s disease [40] but also to cognitive deficits—lower arousal, reduced attention [11] and memory deficits [41]—that are typical in old age. Indeed, in older individuals, locus coeruleus integrity was associated with better memory performance [42].

We also asked whether decreased neuronal number leads to a loss of noradrenergic axons or whether with increasing arborization the remaining neurons can maintain the original axonal network by sprouting axonal projections as observed in Alzheimer´s disease patients [43]. Our work now suggests that, in ageing mice, lower neuronal numbers are associated with a reduced density of noradrenergic terminals, suggesting a low level of compensation. We noted that the reduction of NET-positive axons in the function of TH-positive neurons in the LC is more intensive in *Cnr1*^−/−^ than in *Cnr1*^+/+^ mice. We hypothesize that, in the knockout line, the compensation is even lower (if any) than in wild-type animals. It was previously hypothesized that LC neurons are organized into clusters having unique efferent regions [44]. Our study now suggests that the age-related neuronal loss affects these clusters similarly, because we found similar changes in the density of axon terminals in the projection areas. The decreasing noradrenergic signaling in the efferent regions leads to a decline in synaptic plasticity [45,46], which may contribute to the deficits in learning flexibility. Indeed, in old *Cnr1*^−/−^ mice where we found a reduced number of LC neurons, the learning flexibility was also severely impaired [35].

As a possible explanation for the protective effect of CB1 receptor activity we considered its anti-inflammatory effect on glia cells. Indeed, loss of hippocampal neurons in constitutive or GABAergic neuron specific *Cnr1*^−/−^ mice [35] was associated with increased pro-inflammatory glial activity. However, we found no difference in microglial Iba1, TNFα levels or size of GFAP-positive astrocytes covered areas in the LC between old *Cnr1*^−/−^ and wild-type mice. Although microglia numbers were enhanced in *Cnr1*^−/−^ mice, which is generally interpreted as a sign of inflammation, the normal Iba1 and TNFα levels suggest that these microglias were not more pro-inflammatory. Thus, it is unlikely that increased pro-inflammatory glial activity is responsible for the loss of LC neurons in *Cnr1*^−/−^ mice.

The reason why noradrenergic neurons in the locus coeruleus seem to be more vulnerable to ageing is not fully known. It has been suggested that the combination of several factors is responsible for the neuronal loss in this specific neuronal population: high oxidative stress due to the noradrenaline synthesis, high neuronal iron content, autonomous pacemaking activity and a very high axonal arborization size [47,48]. Importantly, the cumulative effect of these factors could eventually result in a global energetic failure [49] and be responsible for the cell death.

There are several lines of evidence that cannabinoid system activity is neuroprotective and influences brain ageing [50,51]. Importantly, factors contributing to the vulnerability of LC noradrenergic neurons—increased oxidative stress, pacemaking activity, impaired proteostasis due to the high arborization, and high load on mitochondria due to the big energetic need—are all influenced by the cannabinoid system.

Cannabinoids are known to possess antioxidant-like properties [52] through the CB1 receptor-dependent [53] and independent mechanism [54]; therefore, the level of antioxidant defense may correlate with the cannabinoid signaling activity. Cannabinoids might also influence proteostasis in LC neurons partly by increasing lysosomal stability and integrity [55,56] and partly by modulating mTOR signaling [57,58]. Activation of CB1 receptors on the neuronal membrane decreases firing frequency and protects against depletion of energy sources, whereas activation of mitochondrial CB1 receptors decreases mitochondria activity, thus enabling a coupling between firing activity and energy need of the neurons [59]. Moreover, CB1r agonists decrease oxygen consumption, ROS production [60], and oxidative phosphorylation [61], and, under cellular stress, cannabinoids protect mitochondria [62], which together could be essential for the survival of noradrenergic neurons. Cellular stress resistance is largely dependent on cell metabolism and also on mitochondrial function. The biogenesis and dynamics of mitochondria is controlled by three major nutritional sensors: mTOR, AMPK and sirtuins [63]. Interestingly, cannabinoid system activity influences each of these controlling pathways. Activation of CB1 receptors upregulates mTOR signaling [57] and the activity of AMPK [64] in the brain, and there is a mutual interaction between the cannabinoid and sirtuin signaling. These studies and the observation that the mitochondria in neurons of *Cnr1*^−/−^ mice show an aberrant morphology [65] together suggest that CB1r on mitochondria can play a significant role in the neuroprotective effect of cannabinoid system activity. Further experiments are necessary to clarify whether the protective effect of cannabinoid system activity on LC noradrenergic neurons is cell intrinsic or extrinsically mediated by CB1 receptors on afferent neurons or on glia cells. For that, specific targeting of noradrenergic neurons is necessary where the expression of Cre is specific to the dopamine beta hydroxylase (DBH·)-positive noradrenergic neurons.

As a summary, we observed that there is a significant reduction in the number of the ageing-sensitive LC noradrenergic neurons in *Cnr1*^−/−^ mice. On the other hand, the number of SN and VTA dopaminergic neurons is not influenced by ageing in wild-type animals and also not by the genetic deletion of CB1 receptor. Therefore, we hypothesize that the increased neuronal loss in the LC of *Cnr1*^−/−^ mice is a result of an accelerated brain ageing due to the lack of neuroprotective effect of CB1 receptor activity.

## 4. Materials and Methods

### 4.1. Animals

We used two cohorts of male mice on a congenic C57BL6/J background bred at the animal facility of the Medical Faculty at the University of Bonn. The first cohort contained 14 3- and 22-month-old wild-type mice, and the second cohort contained 10 3- and 18-month-old *Cnr1*^−/−^ (B6.cg Cnr1 tm1Zim) and 12 age-matched wild type littermates. Animals were housed under a reversed light cycle in groups of 3–5 with food and water ad libitum. Animal experiments were approved by the Landesamt fuer Natur, Umwelt und Verbraucherschutz Nordrhein-Westfalen (LANUV NRW; 84-02.04.2015.A265).

### 4.2. Tissue Preparation

The animals were deeply anesthetized with ketamine and xylazine and transcardially perfused with ice-cold phosphate buffered saline (PBS) followed by 4% formaldehyde solution between 10 am and 2 pm. The isolated brains were post-fixed 2 h in 4% formaldehyde solution, kept in 20% sucrose overnight for cryoprotection, snap frozen in dry ice-cooled isopentane and stored in −80 °C. Afterwards, 18 μm thick coronal slices were serially sectioned using a cryostat (CM3050 S, Leica, Wetzler, Germany) and mounted on glass slides. Glass slides were kept at −80 °C until further use.

### 4.3. Microscopy

Frozen sections were dried for 30 min at 37 °C on a hot plate. After drying, the slices were framed with a PapPen, washed in PBS and permeabilized in PBS containing 0.5% Triton X-100 for 1 h. Nonspecific binding was blocked by incubation in PBS containing 3% bovine serum albumin (BSA, PAN Biotech, Jabalpur, India) for 2 h. Next, slices were incubated overnight at 4 °C with the primary antibody: sheep anti-TH (1:1000, Abcam, Cambridge, UK), mouse anti-TNFα (1:100, Abcam, Cambridge, UK) rabbit anti-Iba1 (1:2000, Wako, Osaka, Japan) or chicken anti-GFAP (1:300, Abcam, Cambridge, UK) diluted in PBS containing 0.5% BSA and 0.05% Triton X-100. Afterwards, slides were washed three times in PBS, followed by incubation with the respective secondary antibody (AF488 anti-sheep, AF647 anti-mouse, AF647 and AF568 anti-rabbit and AF647 anti-chicken, all 1:1000 all from Life Technologies, Darmstadt, Germany) in PBS containing 0.5% BSA and 0.05% Triton X-100 for 2 h. Then, slides were washed in PBS, briefly immersed in MilliQ water, mounted with 4’,6-diamidino-2-phenylindole (DAPI, Southern Biotecnology Associates, Birmingham, AL, USA) and covered and stored at 4 °C. For the NET staining, TBS instead of PBS has been used and the slices were subjected to antigen retrieval with citrate buffer for 20 min at 65 °C. The primary antibody (rabbit anti-NET, 1:2000, Synaptic Systems, Gottingen, Germany) and the secondary antibody (AF647 anti-rabbit, 1:1000, Life Technologies, Darmstadt, Germany) were also diluted in TBS containing 3% BSA and 10% donkey/goat serum. Images were obtained with an LSM SP8 confocal microscope (Leica, Wetzler, Germany).

For light microscopy, the processing of the tissues was identical, but we used biotinylated donkey anti-sheep secondary antibody (1:500, Abcam, Cambridge, UK) as a. Slides were incubated with ABC-reagent (Vectastain, Vector Laboratories, CA, USA) for 30 min and immersed in 0.5 mg/mL diaminobenzidine (DAB) and 0.5 mg/mL NH_4_Ni-Sulphate in 50 mM Tris pH 7.3. The reaction was started with H_2_O_2_ and stopped by washing the slides in 50 mM Tris. Subsequently, slides were rinsed in MilliQ water and dehydrated with serial incubations in solutions with increasing concentrations of ethanol and xylol. The slices were mounted with Roti Histokitt II mounting medium (Carl Roth GmbH, Karlsruhe, Germany), covered and stored at 4 °C. Images were obtained with Axio Imager M2 microscope (Zeiss, Oberkochen, Germany) with 20 x objective lens.

For the stereological quantification of TH-positive cells, every 4th slice of the region of interest was collected for a total of 8–10 slices per sample. Then, we stained for TH immunoreactivity. The total number of TH-positive neurons in both hemispheres was estimated manually using the plugin cell counter from Fiji software (Ver. 2.1.0/1.53c, NIH, Bethesda, MD, USA).

Iba1 and TNFα signal intensities were analyzed within the Iba1-positive microglia within the LC in both hemispheres using Fiji software. GFAP staining was analyzed as the percentage of GFAP-covered area in the LC.

The density of NET-positive axons (as % area covered by NET-positive signal) was analyzed in the parietal cortex, basolateral amygdala, CA1, CA3 and dentate gyrus regions of the hippocampus and in the mediobasal hypothalamus in both hemispheres.

### 4.4. Determination of Noradrenaline Levels

Noradrenaline levels were quantified in regions representing the main output areas (hippocampus, basolateral amygdala, parietal cortex, mediobasal hypothalamus) of LC in 18-month-old male *Cnr1*^+/+^ and *Cnr1*^−/−^ mice. Mice were deeply anaesthetized by isoflurane inhalation and transcardially perfused with ice-cold PBS. Brains were quickly removed and stored at –80 °C until analysis. Brain regions of interest were isolated using the punch technique from both hemispheres of the frozen brain tissue and homogenized on ice in 0.01 N HCl, 0.15 mM EDTA and 4 mM sodium metabisulfite. Protein concentration was quantified using the Pierce™ BCA Protein Assay Kit (Thermo Fisher Scientific, Waltham, MA, USA). Quantification of noradrenaline was performed using the Mouse/Rat Noradrenaline (Norepinephrine) ELISA Assay Kit (Eagle Biosciences, Inc., Amherst, NH, USA). For extraction, 40 µg of protein per sample were used in a total volume of 400 µL. Extracted samples were eluted in 250 µL 0.025 M HCl and split into 100 µL duplicates for the subsequent enzyme and ELISA procedure. A total of 20 µL of standards and controls were extracted in a total volume of 400 µL and processed in duplicates.

### 4.5. Statistics

The number of the animals or samples is indicated in the figure legends. All the data are presented as means ± SEM and statistical analysis was done using the Prism software (Ver. 9.0.0., GraphPad Software, San Diego, CA, USA) Data distribution was analyzed using the D’Agostino and Pearson normality test. Statistical significance was determined by Student *t*-test, Mann–Whitney test or 2-way ANOVA. Significant outliers were identified and excluded by using Grubb’s test.

## Figures and Tables

**Table 1:** Correlation analysis between the number of tyrosine hydroxylase (TH)-positive cells and the NET-positive axon densities in the parietal cortex (Pa CTX), basolateral amygdala (BLA), mediobasal hypothalamus (Mb HY) and hippocampal cornu ammonis 1 (HC CA1), cornu ammonis 3 (HC CA3), and dentate gyrus (HC DG) in wild-type and *Cnr1*^−/−^ mice.

Brain Area	Wild-Type		*Cnr1* ^−/−^	
	Correlation (r, Spearman)	Significance	Correlation (r, Spearman)	Significance
Pa CTX	0.6399	0.0171	0.6857	0.0214
BLA	0.4502	0.0685	0.2713	0.2894
Mb HY	0.6643	0.0137	0.7682	0.0096
HC, CA1 region	0.5527	0.0345	0.8316	0.0042
HC, CA3 region	0.8863	0.0005	0.6826	0.022
HC, DG region	0.7701	0.0042	0.3857	0.1366

## Data Availability

The data presented in this study are available on request from the corresponding author.

## Abbreviations

CB1r	cannabinoid receptor type-1
*Cnr1* ^−/−^	cannabinoid 1 receptor knockout
GFAP	glial fibrillary acidic protein
Iba1	ionized calcium-binding adapter molecule 1
LC	locus coeruleus
NE	noradrenaline
NET	noradrenaline transporter
SN	substantia nigra
TH	tyrosine hydroxylase
TNFα	tumor necrosis factorα
VTA	ventral tegmental area
Pa CTX	parietal cortex
BLA	basolateral amygdala
Mb HY	mediobasal hypothalamus
HC	hippocampus
CA1	cornu ammonis 1
CA3	cornu ammonis 3
DG	dentate gyrus
mTOR	mechanistic target of rapamycin
AMPK	5’ adenosine monophosphate-activated protein kinase
PBS	Phosphate buffered saline
